# Hyperandrogenism and Metabolic Syndrome Are Associated With Changes in Serum-Derived microRNAs in Women With Polycystic Ovary Syndrome

**DOI:** 10.3389/fmed.2019.00242

**Published:** 2019-11-01

**Authors:** Anja E. Sørensen, Pernille B. Udesen, Grzegorz Maciag, Julian Geiger, Negar Saliani, Andrzej S. Januszewski, Guozhi Jiang, Ronald C. Ma, Anandwardhan A. Hardikar, Marie Louise M. Wissing, Anne Lis M. Englund, Louise T. Dalgaard

**Affiliations:** ^1^Department of Science and Environment, Roskilde University, Roskilde, Denmark; ^2^Odense University Hospital, The Danish Diabetes Academy, Odense, Denmark; ^3^Fertility Clinic, Department of Gynecology and Obstetrics, Zealand University Hospital, Køge, Denmark; ^4^Diabetes and Islet Biology Group, NHMRC Clinical Trials Centre, University of Sydney, Camperdown, NSW, Australia; ^5^Department of Medicine & Therapeutics, Faculty of Medicine, The Chinese University of Hong Kong, Shatin, Hong Kong

**Keywords:** microRNA, polycystic ovary syndrome, serum free testosterone, hyperandrogenism, metabolic syndrome, TaqMan low density arrays

## Abstract

Polycystic ovary syndrome (PCOS) remains one of the most common endocrine disorder in premenopausal women with an unfavorable metabolic risk profile. Here, we investigate whether biochemical hyperandrogenism, represented by elevated serum free testosterone, resulted in an aberrant circulating microRNA (miRNAs) expression profile and whether miRNAs can identify those PCOS women with metabolic syndrome (MetS). Accordingly, we measured serum levels of miRNAs as well as biochemical markers related to MetS in a case-control study of 42 PCOS patients and 20 Controls. Patients were diagnosed based on the Rotterdam consensus criteria and stratified based on serum free testosterone levels (≥0.034 nmol/l) into either a normoandrogenic (*n* = 23) or hyperandrogenic (*n* = 19) PCOS group. Overall, hyperandrogenic PCOS women were more insulin resistant compared to normoandrogenic PCOS women and had a higher prevalence of MetS. A total of 750 different miRNAs were analyzed using TaqMan Low-Density Arrays. Altered levels of seven miRNAs (miR-485-3p, -1290, -21-3p, -139-3p, -361-5p, -572, and -143-3p) were observed in PCOS patients when compared with healthy Controls. Stratification of PCOS women revealed that 20 miRNAs were differentially expressed between the three groups. Elevated serum free testosterone levels, adjusted for age and BMI, were significantly associated with five miRNAs (miR-1290, -20a-5p, -139-3p, -433-3p, and -361-5p). Using binary logistic regression and receiver operating characteristic curves (ROC), a combination panel of three miRNAs (miR-361-5p, -1225-3p, and -34-3p) could correctly identify all of the MetS cases within the PCOS group. This study is the first to report comprehensive miRNA profiling in different subgroups of PCOS women with respect to MetS and suggests that circulating miRNAs might be useful as diagnostic biomarkers of MetS for a different subset of PCOS.

## Introduction

Polycystic ovary syndrome (PCOS) presents with a multifactorial etiology that includes genetic predisposition (1) and environmental factors. PCOS is the most common endocrine disturbance in women and has a prevalence of 6.1–19.9% among women in the reproductive-age (2). According to the Rotterdam criteria, PCOS is diagnosed, when two out of the three following criteria are fulfilled: (1) Oligo- or anovulation, (2) Clinical and/or biochemical signs of hyperandrogenism, and (3) Polycystic ovaries and exclusion of other etiologies (such as congenital adrenal hyperplasia, androgen-secreting tumors, Cushings' syndrome) (3–5).

Elevated androgen levels constitute a key feature in the pathogenesis of PCOS (5) with high testosterone (T) levels being linked with abdominal fat distribution (6), glucose intolerance (5), and insulin resistance (5) as well as ovulatory dysfunction (7). Both androstenedione and T contribute to the total circulating androgen pool. A large cohort study of PCOS women showed that elevated free T levels conferred increased metabolic risk compared to PCOS women with isolated increased androstenedione (8). Normoandrogenic oligo- or anovulatory patients with polycystic ovaries often have a milder metabolic profile that more closely resembles that of control subjects (9, 10). The clinical indication of hyperandrogenism, scored by Ferriman Gallwey (FG), varies by ethnicity, sensitivity of the hair follicles to androgens (11) as well as the biochemical level of circulating androgens. Thus, FG-score correlates only modestly with total serum T (12). Furthermore, hyperandrogenemia, but not hirsutism, was found to be an independent predictor of metabolic syndrome (MetS) presence in PCOS patients (13). Given that PCOS women already have an unfavorable metabolic profile in early adulthood, there is a need for PCOS patient stratification in terms of metabolic risk outcomes independently of the different PCOS phenotypes.

Circulating microRNA (miRNA) profiles may constitute valuable screening tools as non-invasive biomarkers as they are abundant in serum, resistant to nucleases and stable over freeze-thaw cycles. Sensitive and specific reverse-transcription polymerase chain reaction (RT-qPCR)-based assays for their detection suggest miRNAs as promising biomarker candidates (14). In cells, miRNAs post-transcriptionally impair mRNA translation and, consequently, a dysregulated miRNA expression profile may affect various cellular processes and pathways, in keeping with the complexity of PCOS (15). Moreover, alterations in the circulating miRNA profile may reflect underlying changes in miRNA expression in or secretion from several tissues.

Research concerning miRNAs in serum of PCOS patients is limited; at the present time, few studies examine this topic (16–24). There is consensus that a miRNA profile has the ability to distinguish between PCOS patients and controls. However, there is only limited agreement on which miRNAs that could serve as markers of PCOS (15), although miR-21 and miR-93 have received attention by several groups as being involved in the pathogenesis of PCOS (25–31). Circulating miRNAs may also (in time, given further investigations) present as valuable informants about various pathophysiological processes occurring in remote and potentially inaccessible tissues.

In view of these findings, the present study evaluates, in an unbiased and comprehensive fashion, how miRNAs in serum relate with PCOS in Danish women. Furthermore, because of the significant role of hyperandrogenemia in many aspects of PCOS, patients were categorized into biochemical hyperandrogenic or normoandrogenic PCOS groups to discover more precise links between different phenotypes of PCOS and the level of circulating of miRNAs. In this study, we have systematically screened serum miRNAs from 62 study participants using the TaqMan Low-Density Array (TLDA) of 750 different miRNAs and determined their correlation with clinical and metabolic indices found in PCOS women (*n* = 42) compared with age as well as in BMI matched Controls (*n* = 20). Previous studies have used triage-based study designs that investigated RNA from a pool of PCOS patients or only a subset of study participants for TLDA or sequencing, followed by a validation of selected miRNAs by individual RT-qPCR in a larger study sample (16, 32). To our knowledge, this is the first study with a comprehensive screening of an entire PCOS study population for this number of different miRNAs.

## Materials and Methods

### Study Population

During January 2010–February 2013, we recruited 42 women diagnosed with PCOS according to the Rotterdam 2003 criteria and 20 healthy, age, and body mass index (BMI) matched, regular cycling women who were referred to the fertility clinic due to tubal factor infertility or male infertility. For evaluation purposes, PCOS women were subdivided according to high or normal free serum T levels. A PCOS patient with free serum T above 0.034 nmol/l was considered biochemical hyperandrogenic according to Danish reference intervals (95th percentile) for serum free T for females aged 11–50 (33, 34). Further details of the population is given elsewhere (26).

### Ethics

This study was approved by the Local Scientific Ethical Committee of Region Zealand, DK (approval no. SJ-156). The study was conducted in accordance with the Helsinki Declaration II and all the participants gave written informed consent before their inclusion.

### Baseline Characteristics

Anthropometric and biochemical measurements obtained in this study population have been described previously (26, 35). Serum T, androstenedione, and dehydroepiandrosterone sulfate (DHEAS) were measured by liquid chromatography-tandem mass spectrometry (PerkinElmers Inc., USA), sex hormone binding globulin (SHBG) by an immunometric assay with fluorescence detection on the Abbott Architect i2000 analyzer. Estimation of free serum T was obtained based on SHBG and total T according to Vermeulen et al. (36). Insulin resistance was indirectly assessed using the homeostatic index of insulin resistance (HOMA-IR) index according to this formula: HOMA-IR = [Fasting plasma insulin (mU/L) • Fasting plasma glucose (mmol/L)]/22.5. In accordance with current consensus statements (37), Anti-Müllerian hormone (AMH) was not included in the present study. MetS was present if any three of the following criteria was fulfilled according to International Diabetes Federation consensus statement (38): (1) waist circumference ≥80 cm, (2) Elevated blood pressure (≥130/85 mmHg), (3) Raised fasting blood glucose ≥5.6 mmol/L, (4) Decreased HDL cholesterol (<1.3 mmol/L), and (5) Increased triglyceride levels (≥1.7 mmol/L).

### Sample Preparation and Isolation of miRNA From Serum

Total RNA was extracted from serum samples with Tri-reagent LS (Sigma-Aldrich, Brøndby, Denmark), according to manufacturers' protocol and stored at −80°C until analysis. RNA concentration and purity were assessed using a NanoDrop ND-1000 (Thermo Fisher Scientific, Hvidovre, Denmark).

### MiRNA Serum Profiling

Megaplex RT-primer pools were used to reverse transcribe 125 ng RNA to cDNA and TaqMan MicroRNA Reverse Transcription Kit (ThermoFisher Scientific) according to manufacturers' protocol. The RT-product was pre-amplified with TaqMan® PreAmp Master Mix and miRNA PreAmp primer pools. Pre-amplified samples were diluted with 0.1 × TE buffer (pH 8.0) and stored at −80°C until analysis. The miRNA profile was acquired using Human TaqMan Low-Density Array (TLDA) cards v.3 containing 750 unique miRNAs (ThermoFisher Scientific). All of the TLDA cards had the same lot number to minimize variation. Expression data were obtained using the ViiA 7 real-time PCR system and analyzed using QuantStudio software. Amplification plots were manually inspected and assays with poor or no amplification (undetectable) were excluded from further analysis (*n* = 406). A pre-specified criterion was that a miRNA needed to be present in at least 10 (15%) of the samples, so as to be included in further analyses. Data were normalized using the global mean normalization method (39). Data from this miRNA discovery profiling would be available upon reasonable request.

### Technical Validation of miRNA by Individual qPCR

A total of 200 ng serum RNA was reverse transcribed with mature miRNA-specific stem-loop RT-primers using SuperScript III (Thermo Fisher Scientific). Subsequently, qPCR was performed on diluted cDNA using QuantiTect SYBR Green PCR master mix (Qiagen, Copenhagen, Denmark) as described previously (26) (oligos are listed in Supplementary Table 1). All reactions were performed in duplicate. The miRNA levels were normalized to the expression of miR-484, which showed minimal variation across samples and experimental groups according to the NormFinder (40) algorithm (Supplementary Figure 1). MiRNA-484 has been used by others as a reference and normalization miRNA (41). This also provided a validation of our TaqMan (TLDA) data using SYBR Green qPCR chemistry.

### Statistical Analyses

Statistical analyses were performed using the Statistical Packages for Social Sciences (SPSS) vers. 24 or the statistical package R and R-Studio vers. 3.1.3. A prior power analysis with the given sample size shows that we have a power of more than 90% to detect a fold change of 1.5 with a relative SD of 0.4 with a Student's *t*-test (estimated effect size, Cohen's d, 1.25).

TLDA miRNA relative levels were quantified by the 2^−ΔΔ*Ct*^ method. Validation of the relative expression of individual miRNAs was quantified by the standard curve method. Normal distribution was assessed by a Kolmogorov–Smirnov test. Logarithmic transformation was applied to the non-normally distributed data. Group means were compared by Student's *t*-test or one-way ANOVA with Tukey's *post-hoc* test (two-sided). Values were reported as mean ± SD or medians (interquartile range) as appropriate. Pearson correlations were visualized using the R package corrplot (42). Partial Pearson correlations were performed to control for the effects of age and BMI. A volcano plot was plotted to display fold changes (log_2_) against statistical *t*-test *p*-values (–log_10_) for each miRNA in relation to PCOS women and Controls. To adjust for the potentially confounding effect of age and BMI, binary logistic regression analyses for the association between each of the miRNA and PCOS or multinomial logistic regression for the three different groups were carried out represented by the odds ratio (OR) and 95% confidence interval (CI).

A *p*-value of < 0.05 was considered statistically significant. Adjustment for multiple testing was not found applicable given the lack of independence of variables (43) [e.g., miRNAs show a high degree of correlation within groups (44)] in the dataset and the exploratory nature of the study. GraphPad Prism vers. 8 (GraphPad Inc., La Jolla, CA, U.S.A.) was used for figure preparation.

## Results

### Demographics, Clinical and Biochemical Markers of the Study Population

Both PCOS women and Controls were well matched for age and BMI (Table 1). In the PCOS group FG scores, androgens, luteinizing hormone (LH) were significantly elevated (Table 1). The biochemical hyperandrogenic PCOS women were more insulin resistant (*p* < 0.01) compared to the normoandrogenic PCOS group. Furthermore, the incidence of MetS was higher in PCOS women with elevated serum free T (*p* < 0.05).

**Table 1:** Baseline characteristics of study participants.

	**Control**	**PCOS**	***p***	**Normo PCOS**	**Hyper PCOS**	***p***
*N*	20	42		23	19	
Age	27 (7.5)	27 (6.3)	NS	29 (7)	27 (6)	NS
Height (cm)	166.9 ± 6	169.5 ± 6.1	NS	171.2 ± 6.4	167.3 ± 5.2	NS
Weight (kg)	71.3 (23.8)	72.3 (18.1)	NS	71.6 (15)	75 (16.2)	NS
Body Mass Index (kg/m^2^)	25.0 (6.9)	24.4 (5.9)	NS	23.8 (4.7)	28.4 (6.2)	NS
Waist-to-hip ratio	0.8 ± 0.1	0.8 ± 0.1	NS	0.8 ± 0.1	0.8 ± 0.1	NS
Ferriman–Gallwey (FG) score	1.0 (1.0)	4.0 (6.3)	<0.01	4.0 (6.0)	5.0 (6.0)	<0.01^a^^,^^b^
Total testosterone (T) (nmol/L)	1.1 (0.5)	2.4 (1.5)	<0.01	1.9 (1.1)	3.7 (2.3)	<0.01^a^^,^^b^^,^^c^
Free testosterone (T) (nmol/L)	0.014 (0.005)	0.025 (0.045)	<0.01	0.020 (0.006)	0.066 (0.018)	<0.05^a^^,^^b^^,^^c^
Sex hormone binding globulin (SHBG) (nmol/L)	71.0 (36.3)	65.5 (55.8)	NS	88.0 (53)	46.0 (36)	<0.01^a^^,^^c^
Androstenedione (nmol/L)	4.4 (2.5)	7.7 (4.5)	<0.01	5.4 (2.5)	10.1 (4.3)	<0.01^a^^,^^b^^,^^c^
Dehydroepiandrosterone sulfate (DHEAS) (nmol/L)	5,291 ± 1,949	5,959 ± 2,473	NS	4,642 ± 1,644	7,554 ± 2,393	<0.01^a^^,^^c^
Follicle-stimulating hormone (FSH) (IU/L)	6.3 ± 1.9	5.6 ± 1.7	NS	5.7 ± 1.9	5.4 ± 1.5	NS
Luteinizing hormone (LH) (IU/L)	5 (3.8)	11.6 (5.9)	<0.01	10.8 (6.6)	12 (6.3)	<0.01^a^^,^^b^
Estradiol (nmol/L)	0.19 (0.04)	0.2 (0.03)	NS	0.2 (0.12)	0.2 (0.01)	NS
Prolactin (mIU/L)	212.0 (132)	212.0 (135)	NS	237.0 (141)	209.0 (118)	NS
Thyroid stimulating hormone (TSH)	1.8 (1.2)	1.7 (1)	NS	1.7 (0.7)	1.5 (1.2)	NS
Alanine transaminase (ALAT) (u/L)	15.5 (10)	18 (16.5)	NS	17 (8)	22 (19)	NS
Fasting plasma glucose (mmol/L)	5.0 (0.5)	5.1 (0.5)	NS	5.1 (0.4)	5.2 (0.6)	NS
Fasting serum insulin (mU/L)	7.7 (5.5)	6.9 (8.1)	NS	6.2 (5.6)	8.8 (8.8)	<0.01^c^
Fasting plasma C-peptide (pmol/L)	605(260)	560 (390)	NS	470 (270)	680 (390)	<0.05^a^^,^^c^
HOMA-IR (mU·mmol·L^−2^)	1.8 (1.3)	1.5 (1.9)	NS	1.3 (1.5)	2.1 (2.1)	<0.01^c^
Total cholesterol (mmol/L)	4.4 (1.1)	4.3 (0.9)	NS	4.4 (1.3)	4.3 (0.7)	NS
LDL cholesterol (mmol/L)	2.6 ± 0.8	2.4 ± 0.7	NS	2.5 ± 0.7	2.4 ± 0.7	NS
HDL cholesterol (mmol/L)	1.6 (0.4)	1.5 (0.5)	NS	1.6 (0.5)	1.4 (0.4)	<0.05^a^^,^^c^
Triglycerides (mmol/L)	0.6 (0.2)	0.7 (0.5)	NS	0.6 (0.3)	1.0 (0.8)	<0.05^a^^,^^c^
Metabolic syndrome (yes/no)	1/20	7/35	NS	1/22	6/13	<0.05^#^

*Baseline characteristics of the study participants. Data presented as mean ± SD or as median (interquartile range) if not normally distributed. Biochemical hyperandrogenic (Hyper) and normoandrogenic (Normo) PCOS patients were defined based on a free T level above or below 0.034 nmol/L, respectively. HOMA-IR, homeostatic model assessment of insulin resistance; HDL, high-density lipoprotein; LDL, low-density lipoprotein; NS, not significant*.

a *Significant difference between hyperandrogenic patients and Controls after Tukey post-hoc*.

b *Significant difference between normoandrogenic patients and Controls after Tukey post-hoc*.

c *Significant difference between hyperandrogenic patients and normoandrogenic after Tukey post-hoc*.

# *Significant difference between hyperandrogenic patients and both normoandrogenic PCOS women and Controls after Fisher's Exact test. P < 0.05 were consider significant*.

### Human Serum miRNA Profiles by Array Analysis

We performed an extensive qPCR based profiling of 750 different miRNAs in serum from all 62 subjects in our cohort in order to identify dysregulated miRNAs in PCOS women. Among the predefined 750 miRNAs, we identified 303 miRNAs (40.4%) as robustly present in serum. The 10 most abundant serum miRNAs in the entire study population had similar levels with miR-518 species being most abundant among circulating miRNAs (C_T_ ≤ 10.0, Supplementary Table 2). Analyzing the intercorrelation between the 50 most highly expressed miRNAs revealed that the majority (*n* = 41) of the miRNAs correlated with each other belonging either in one large or in one smaller cluster (Supplementary Figure 2). Moreover, we evaluated the variability of the miRNAs in the control women and in the entire sample set (Supplementary Figure 3). The clear majority of the circulating miRNAs had an inter-assay coefficient of variation (CV) of <5%; only 18 out of 303 miRNAs had a CV >5% (Supplementary Figure 3). Thus, although there is interpersonal variation in the levels of each miRNA, with few exceptions, the levels of each species of miRNA appears quite defined, at least in our specific cohort of PCOS women and Controls.

### Circulating miRNAs Are Able to Differentiate Between PCOS Patients and Controls

**Figure 1:**
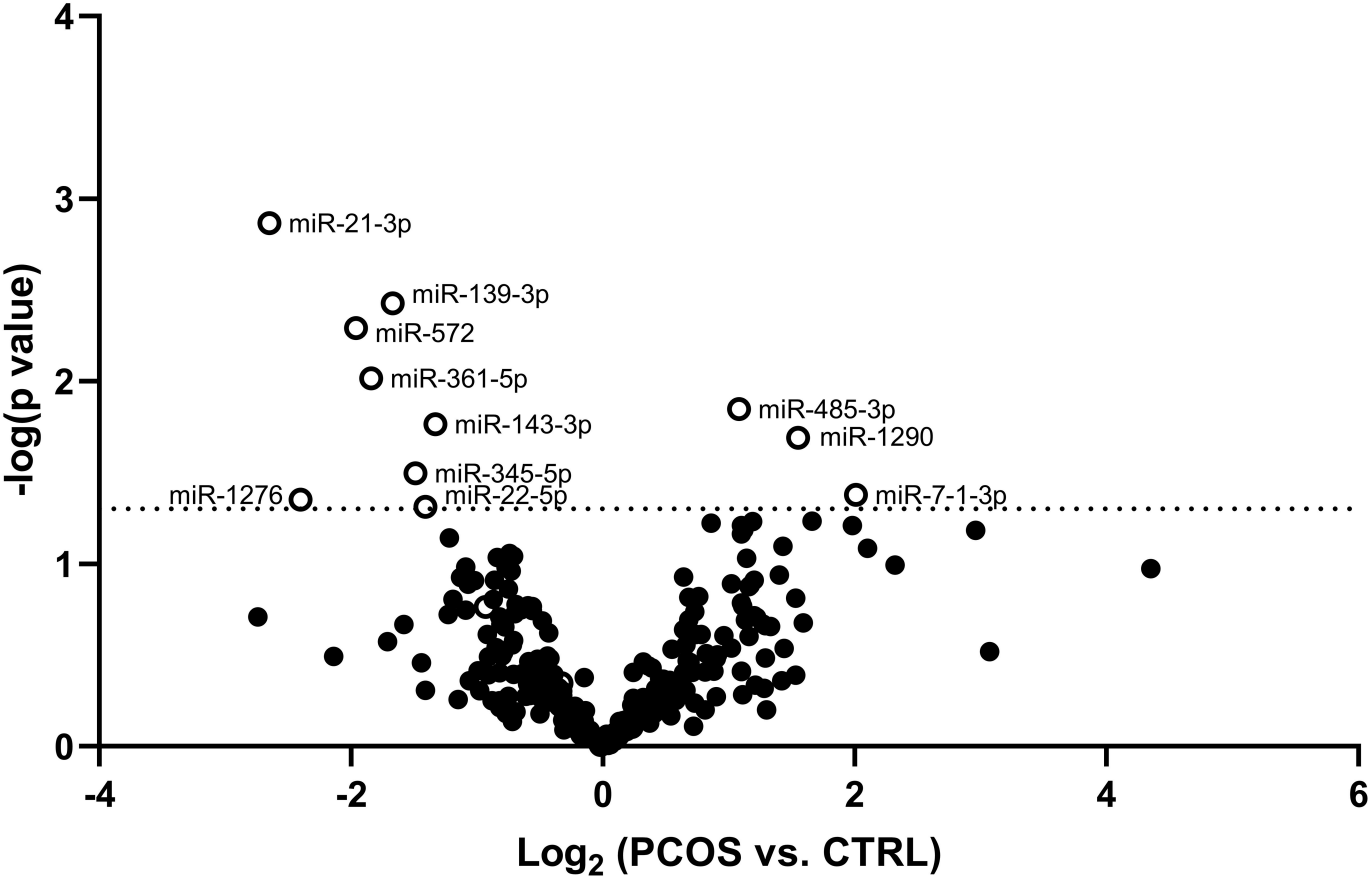
Volcano plot displaying differences in circulating miRNA levels between PCOS women and Controls. Volcano plot based on miRNA mean fold changes between PCOS patients vs. Control subjects. Open circles indicate significantly [*p* < 0.05; –log (*p*-value) of −1.3, dotted line] increased or decreased relative miRNA levels of each miRNA in PCOS patients compared to Control subjects.

**Figure 2:**
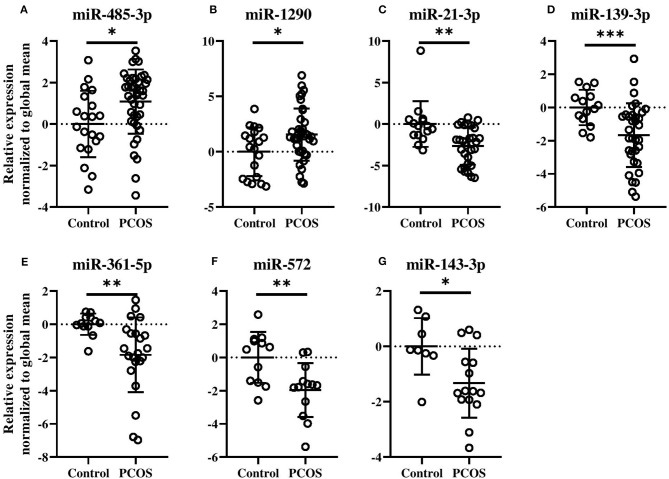
Levels of circulating miRNA species in PCOS patients compared with Control subjects **(A–G)**. Relative fold changes in miRNA quantities normalized to the global mean. Means and standard deviations are displayed. All indicated *p*-values were determined by Student's *t*-test on log_2_ transformed data. Data were adjusted for age and BMI. *p*-value * < 0.05, ** < 0.01, *** < 0.0001.

The potential of each circulating miRNAs (*n* = 303) to differentiate between healthy Controls and women with PCOS was evaluated and is illustrated using a Volcano plot (Figure 1). Three serum miRNAs (1.0% of detected miRNAs; miR-485-3p, miR-1290, and miR-7-1-3p) were significantly increased while eight miRNAs (2.6% of detected miRNAs; miR-21-3p, -139-3p, -572, -361-5p, -143-3p, -345-5p, -1276, and miR-22-5p) were significantly decreased in PCOS women compared with Controls (Figure 1). Half of the miRNAs showed more than an absolute fold difference of 1.8 between the two groups. As obesity amplifies and worsens the metabolic and reproductive abnormalities in PCOS and advanced age gradually decreases the severity of cardinal features of PCOS, data for the eleven significant miRNAs were adjusted for the confounding effects of age and BMI. Both miR-485-3p and miR-1290 remained significantly associated with increased odds of having PCOS (*p* = 0.02, OR 1.5, 95% CI: 1.1–2.2 and *p* = 0.02, OR 1.3, 95% CI: 1.0–1.8) with a 1.1 and 1.6 fold increase observed in PCOS women compared to Controls (Figures 2A,B).

As can be seen in Figures 2C–G, miR-21-3p (*p* = 0.004, OR 0.6, 95% CI: 0.3–0.9), miR-139-3p (*p* = 0.0004, OR 0.5, 95% CI: 0.3–0.9), miR-361-5p (*p* = 0.001, OR 0.4, 95% CI: 0.2–0.9), miR-572 (*p* = 0.005, OR 0.3, 95% CI: 0.1–0.8), and miR-143-3p (*p* = 0.01, OR 0.3, 95% CI: 0.1–0.9) all remained significantly associated with decreased odds of having PCOS. All five miRNAs were decreased by 1.7-fold or more, except miR-143-3p, in PCOS patients compared to Controls. Levels of the remaining four miRNAs can be found in Supplementary Figure 4.

To confirm the results obtained by the arrays a technical validation was performed for miR-485-3p (Supplementary Figure 5). In the arrays, miR-485-3p was elevated in PCOS women (Figure 2A, *p* = 0.004). The individual qPCR showed that miR-485-3p was indeed significantly increased in PCOS patients compared with the Controls (*p* < 0.001).

### Dysregulated Circulating miRNAs in PCOS Women Subsets

**Figure 3:**
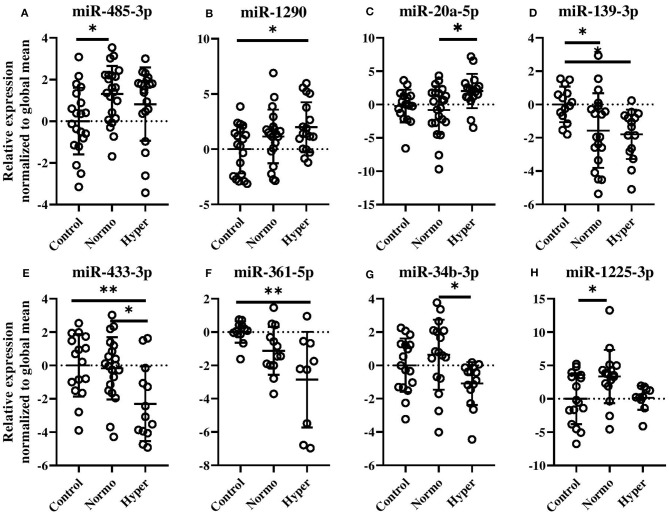
Circulating miRNAs different between healthy, normal cycling Control women, and normo- or hyperandrogenic PCOS patients **(A–H)**. Shown are the relative fold changes of the significantly identified miRNA levels normalized to the global mean, and displayed as relative to the Control group. All indicated *p*-values were determined by one-way ANOVA with a *post-hoc* Tukey test on log_2_ transformed data. Data were adjusted for age and BMI. *p*-value * < 0.05 and ** < 0.01. Normo, biochemical normoandrogenic PCOS; hyper, biochemical hyperandrogenic PCOS.

Evaluation of miRNAs defining the normoandrogenic and hyperandrogenic PCOS patient subsets and Controls showed that 20 miRNAs were expressed differently within the three groups after age and BMI correction (Figure 3 and Supplementary Figure 6). Given that several of the miRNAs showed similar expressions and a high degree of correlation between individual miRNAs, a subset of these are shown in Figures 3A–H. In women with normal free T levels, miR-485-3p was found elevated by 1.3-fold compared to Controls (*p* < 0.05, Figure 3A). Despite also being increased in hyperandrogenic PCOS women, this comparison was not significant. A gradual increase in miR-1290 levels (*p* < 0.05, Figure 3B) as well as a 2-fold increase of miR-20a-5p (*p* < 0.05, Figure 3C) were observed in the hyperandrogenic PCOS women. Circulating miR-139-3p was also significantly decreased in both PCOS subsets compared to healthy Controls with the more hyperandrogenic PCOS women displaying the largest reduction (1.9- vs. 1.7-fold, *p* < 0.05, Figure 3D). Normoandrogenic PCOS women and Controls had similar levels of miR-433-3p while it being 2-fold less abundant in hyperandrogenic PCOS patients compared to either group (*p* < 0.05 or *p* < 0.01, Figure 3E). In comparison to Controls, miR-361-5p decrease gradually the more hyperandrogenic the PCOS women were (2.9-fold, *p* < 0.01, Figure 3F). Both miR-34b-3p and miR-1225-3p were increased in normoandrogenic PCOS patient either compared to hyperandrogenic PCOS women or Controls (*p* < 0.05, Figures 3G,H).

The remaining dysregulated miRNAs between the two PCOS subsets and Controls, which were still significant after adjustments for BMI and age, are displayed in Supplementary Figure 6.

### Circulating miRNAs Correlate With Androgens and Biochemical Markers of Metabolic Syndrome

The relationship between circulating miRNA, androgens and biochemical markers of MetS was explored by correlation analysis controlling for the effects of BMI and age in the entire cohort (Table 2). Hyperandrogenism (in the form of either total T, free T, DHEAS or androstenedione) was positively associated with miR-1290 (r range: 0.47–0.35) and miR-20a-5p (r range: 0.39–0.30). In contrast, miR-139-3p (r range: −0.41 to 0.37), -433-3p (r range: −0.51 to 0.35), -361-5p (r range: −0.48 to 0.34), and -34b-3p (*r* = −0.43 to 0.29) correlated negatively with hyperandrogenism. Furthermore, SHBG, which is generally low in PCOS, was positively correlated with miR-361-5p (*r* = 0.37, *p* = 0.038) and miR-433-3p (*r* = 0.36, *p* = 0.013). Fasting glucose was modestly associated with miR-20a-5p (*r* = 0.32, *p* = 0.022). Both serum insulin (*r* = −0.50) and HOMA-IR (= −0.50) levels correlated negatively with miR-1225-5p (*p* = 0.001). Biochemical markers of MetS such as total cholesterol, HDL cholesterol, and triglycerides were associated with miR-433-3p (*r* = 0.41, *p* = 0.005) and miR-361-5p (HDL: *r* = 0.39, *p* = 0.027, and triglycerides: *r* = −0.37, *p* = 0.036). Lastly, miR-1290 and HDL cholesterol (*r* = −0.28, *p* = 0.042) correlated with each other. Thus, we observed a multitude of correlations between levels of specific circulating miRNAs and features of biochemical hyperandrogenism and the MetS.

**Table 2:** Correlations between biochemical variables and miRNA levels.

**Clinical variable**	**miRNAs**	***r***	***r* adjusted**	***p***	***p* adjusted**
Total T	miR-1290	0.403	0.399	0.002	0.003
	miR-139-3p	−0.426	−0.410	0.003	0.005
	miR-361-5p	−0.369	−0.340	0.032	0.057
	miR-34b-3p	−0.295	−0.286	0.042	0.054
Free T	miR-1290	0.373	0.351	0.005	0.009
	miR-20a-5p	0.364	0.302	0.006	0.028
	miR-139-3p	−0.330	−0.368	0.023	0.013
	miR-433-3p	−0.492	−0.512	0.0004	0.0003
	miR-361-5p	−0.515	−0.461	0.002	0.008
SHBG	miR-20a-5p	−0.289	−0.153	0.031	0.269
	miR-433-3p	0.301	0.358	0.036	0.013
	miR-361-5p	0.419	0.369	0.014	0.038
DHEAS	miR-34b-3p	−0.412	−0.425	0.004	0.003
Androstenedione	miR-1290	0.493	0.468	0.0001	0.004
	miR-20a-5p	0.385	0.393	0.004	0.004
	miR-433-3p	−0.335	−0.353	0.02	0.016
	miR-361-5p	−0.465	−0.478	0.006	0.007
Fasting glucose	miR-20a-5p	0.273	0.317	0.046	0.022
	miR-139-3p	−0.306	−0.262	0.039	0.086
Fasting insulin	miR-20a-5p	0.326	0.177	0.016	0.209
	miR-361-5p	−0.458	−0.312	0.006	0.082
	miR-1225-3p	−0.424	−0.502	0.005	0.001
HOMA-IR	miR-20a-5p	0.347	0.212	0.01	0.132
	miR-361-5p	−0.470	−0.335	0.005	0.061
	miR-1225-3p	−0.427	−0.501	0.005	0.001
Total cholesterol	miR-433-3p	0.336	0.405	0.018	0.005
HDL cholesterol	miR-1290	−0.296	−0.280	0.028	0.042
	miR-361-5p	0.442	0.390	0.009	0.027
Triglycerides	miR-20a-5p	0.401	0.268	0.002	0.052
	miR-361-5p	−0.476	−0.372	0.004	0.036

*Correlation between selected serum miRNAs and baseline characteristics. MiRNA levels (normalized to the global mean) were log_2_ transformed prior to analysis. Some of the biochemical parameters were also log_2_ transformed prior to analysis as described in Materials and Methods. Indicated Pearson correlation coefficients are significant at P < 0.05, p adjusted = Controlling for the effects of age and BMI using partial Pearson correlation analysis. T, Testosterone; SHBG, Sex hormone-binding globulin*.

### Diagnostic Capabilities of Circulating miRNA

**Figure 4:**
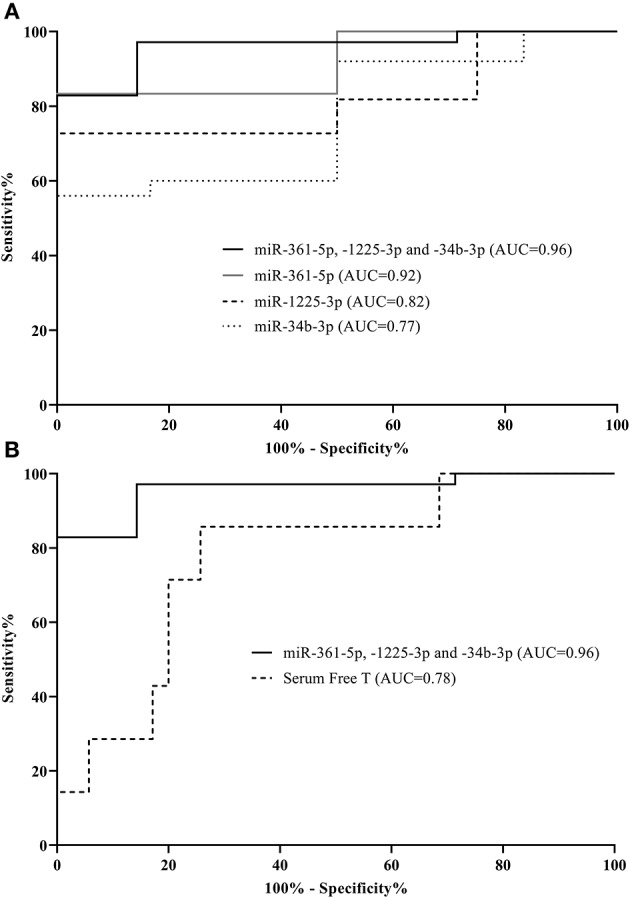
Individual microRNAs are predictive of metabolic syndrome in PCOS women. Receiver operator characteristic curves based on microRNA levels (normalized to global mean). Shown are three microRNAs as well as a combination of these distinguishing between metabolic syndrome cases and those without in the PCOS group **(A)**. Additionally, area under the curves (AUCs) for serum free testosterone compared to the 3-miRNA signature within the PCOS group are displayed **(B)**. The corresponding AUCs and 95% CI: intervals can be found in Supplementary Table 3.

The observed frequency of MetS was significantly higher in the hyperandrogenic PCOS patients, which led us to verify whether potential differences in these 20 circulating miRNAs (Figure 3 and Supplementary Figure 6) could be used to distinguish the presence of MetS in PCOS women. Initially, receiver operator characteristic (ROC) curves were constructed and area under the curve (AUC) values for each miRNA were evaluated (Supplementary Table 3). For PCOS women, as shown in Figure 4A, miR-361-5p was the best independent predictor of MetS (AUC 0.92, 95% CI 0.79–1.0) followed by miR-1225-3p (AUC 0.82, 95% CI 0.65–0.99) and miR-34b-3p (AUC 0.77, 95% CI 0.58–0.96). A biomarker panel was constructed using a combination of the three miRNAs and binary logistic regression analysis. The 3-miRNA panel had an AUC of 0.96 (95% CI 0.90–1.0) and correctly classified all of the seven cases of MetS found in the PCOS group.

Serum free T was significantly elevated in PCOS women with MetS compared to PCOS women without MetS [mean (SD):0.069 (0.034) vs. 0.036 (0.024), *p* = 0.004]. We therefore also investigated the ability of serum T to predict MetS among PCOS women. The discriminatory ability of serum free T alone in relation to MetS in PCOS women was found to be specific (100% specificity), but also to display a low sensitivity of just 14% yielding an AUC of 0.78 (95% CI 0.60–0.96) (Figure 4B). The low sensitivity was observed, because only six out of the twenty hyperandrogenic PCOS women had MetS. Addition of serum free T in the logistic regression model did not significantly improve the AUC of the model and was therefore not included in the combined model (data not shown). Further, the miRNA profile between the two PCOS subsets (normoandrogenic vs. hyperandrogenic) was markedly different (Supplementary Figure 7), which underlines the heterogeneity in phenotypes among women with PCOS.

## Discussion

To date, this is the largest explorative study of serum miRNA profiles in PCOS patients and Controls. Its major strength is the employment of comprehensive array measurements in all individual samples from the study population. Using this approach, we identified two (miR-485-3p and miR-1290) circulating miRNAs with increased levels and five miRNAs (miR-21-3p, -139-3p, -361-5p, -572, and -143-3p) with lower levels in PCOS patients compared to control subjects independent of age and BMI. Furthermore, twenty miRNAs; eight of which were further analyzed (miR-20a-5p, -34b-3p, -139-3p, -361-5p, -433-3p, -485-3p, -1225-3p, and miR-1290) had different levels between either the two PCOS subset or between healthy Controls and one of the PCOS subsets (Figure 3 and Supplementary Figure 6). Additionally, clinical variables related to PCOS, such as free T, DHEAS, and androstenedione were correlated with the miRNAs suggesting their involvement in the pathophysiology of PCOS and its intermediary phenotypes (Table 2). We also observed that five miRNAs (miR-20a-5p, 1225-3p, -433-3p, -1290, and -361-5p) were associated with either aberrant glucose homeostasis or dyslipidemia. Importantly, a 3-miRNA panel consisting of miR-361-5p, -1225-3p, and miR-34b-3p was able to discriminate between MetS in PCOS women independently of androgen status in terms of serum free T.

Since qPCR arrays were performed in all individuals, which to our knowledge has not been done before in relation to PCOS, we were able to determine that a high degree of correlation exists between individual miRNAs (absolute Pearson correlation coefficients ≥0.7, Supplementary Figure 2) suggesting a miRNA:miRNA network and potential co-regulation of certain miRNAs or a common cellular origin of most of the circulating miRNAs. Also, in our specific cohort of PCOS women and Controls, most of the miRNAs displayed a low degree of inter-individual variability, which allows for relatively good power to detect miRNAs that are different between PCOS and control women (Supplementary Figure 3). In recent years, miRNAs have gained increasing attention based on their application in biomarker studies and as much as 30 different serum miRNAs have been identified to be involved in PCOS (16–24), combined with an additional four miRNAs in whole blood (29), and miR-93 in plasma (25). However, only a few studies have aimed to identify/characterize miRNAs associating with PCOS subphenotypes.

It is well established that PCOS is associated with defects in insulin secretion and profound insulin resistance (45). The mitochondrial biogenesis master regulator; peroxisome proliferator-activated receptor γ coactivator 1α (PGC-1α) is required to maintain insulin-mediated signaling as well as mitochondrial function and integrity, thus underscoring its importance in the development of insulin resistance (46). Reduced levels of PGC-1α have been found in PCOS women (47). Of interest, miR-485-3p has been shown to target PGC-1α (48); thus it could be speculated that increased levels of miR-485-3p in PCOS women could inhibit PGC-1α and subsequently impart insulin resistance. Moreover, miR-378a-5p, increased in serum of hyperandrogenic PCOS patients (Supplementary Figure 6B), is located within the gene encoding PGC-1β (PPARGC1B) and causes hepatic insulin resistance by targeting the catalytic subunit of PI3 kinase (49), while simultaneously opposing the actions of PGC-1β (50), suggesting further links between PCOS associated miRNAs and insulin resistance.

Profiling of miRNAs in human follicular fluid identify miR-1290 among the highest expressed miRNAs (51) although not differentially expressed between PCOS women and controls as was observed in the current study. Increased circulating miR-1290 has been reported for various cancers (52, 53) as well as gestational diabetes (54) and non-alcoholic fatty liver disease (NAFLD) (55). Potential predicted targets of miR-1290 are the transforming growth factor β (TGF-β) 2 and 3 as well as the downstream mediators Smad-2,−5,−7, and−9. Dysregulated TGF-β signaling pathway in PCOS pathophysiology has been reported (56–59).

Encoded within the miR-17–92 cluster, miR-20 has been shown to modulate the TGF-β pathway, the E2F transcription family (E2F1-3), the insulin gene enhancer protein (Isl-1), the T-box 1 protein (Tbx1) reviewed by Mogilyansky and Rigoutsos (60); all of which have been linked to PCOS pathophysiology (61).

Out of the seven PCOS-specific miRNAs, miR-21; which was downregulated in our study, has been reported to be increased in serum from PCOS women (23). The observed discrepancy, could partly, be explained by the fact that the included PCOS women in the study by Jiang et al. (23) had a higher BMI compared to controls. Adding to this, obesity was shown by Murri et al. to increase the expression of whole blood miR-21 in PCOS women (29). In support of our findings, reduced serum miR-21 was observed in hyperandrogenic PCOS women, although not significant, by Naji et al. (62) and low plasma miR-21 was demonstrated in type 2 diabetic subjects (63).

Our work revealed a downregulated expression of miR-34b-3p in biochemical hyperandrogenic PCOS women compared to PCOS women with normal serum free T levels (Figure 3). A study on the clinical relevance of miR-34b-3p, although in relation to prostate cancer, revealed that miR-34b-3p could control transcription of the androgen receptor (AR) *in vitro*. Moreover, high tissue expression of AR protein correlated with low miR-34b-3p levels (64). Given the importance of androgens and the androgen receptor in the pathogenesis of PCOS (65), it could be speculated that the low expression of miR-34b-3p observed in the hyperandrogenic PCOS women could enhance expression of AR thereby resulting in dysregulated androgen metabolism. Our results provide a basis for pursuing studies on the functional consequence of altered miRNAs in terms of subphenotypes of PCOS.

Elevated circulating vascular endothelial growth factors (VEGF) may, partly contribute to the highly vascularized and dense hyperechogenic stroma observed in ovaries of PCOS women. Furthermore, insulin can stimulate VEGF secretion while high levels of VEGF have been associated with occurrence and severity of ovarian hyperstimulation syndrome (OHSS) in women with PCOS and negative conception rates augmenting its role in PCOS pathophysiology (66). Low levels of miR-361-5p in our PCOS women; lowest in hyperandrogenic PCOS women (Figures 2, 3), could contribute to increased levels of VEGF-A which is an identified target of miR-361-5p (67).

Prediabetic individuals presented with lower levels of circulating miR-572 compared to newly diagnosed type 2 diabetic patients, although not with lower levels than individuals with normal glucose tolerance (68). It could be speculated whether a decrease in miR-572, as observed in our study, could serve as a biomarker for early detection of prediabetes in PCOS women.

We here demonstrate that serum levels of miR-433-3p significantly differed between hyperandrogenic PCOS women and Controls and normoandrogenic PCOS women (Figure 3). A recent study in pancreatic β-cells showed that miR-433-3p had a protective effect toward glucose toxicity (69) thus low levels of miR-433-3p could contribute to the increased risk of type 2 diabetes observed in PCOS women.

To the best of our knowledge, no studies with relations to PCOS have found a mechanism by which elevated miR-1225-3p or decreased miR-139-3p could be involved in PCOS. Regardless of mammalian species, miR-143 is among the most predominate miRNAs expressed in the ovary (70). Dysregulated miR-143, in mouse models, has been implicated in obesity-associated insulin resistance (71), in the formation of primordial follicles (72) and progesterone release (73).

As stated earlier, miR-93 has received attention from several groups (25, 27, 62, 74). We observe that miR-93-3p is borderline decreased in PCOS women compared to Controls (*p* = 0.051) thus in contrast to increased circulating plasma levels of miR-93 isolated from PCOS women (25). At present, it is unclear whether differences in pre-analytic strategies, mainly normalization, account for the variable findings or whether these findings are due to differences in study populations.

The nature of the current study was to investigate the miRNA profile in serum. We do not know the pathophysiological actions or mechanisms that regulate the serum miRNA profile, nor the tissues from which circulating miRNAs originate. However, it is apparent that a varied tissue distribution of miRNAs exists. The miRNAs present in serum are the result of secretion of these into the circulation by various tissues and cells. Identification of their cellular origin can be difficult. Two studies have compiled a list of miRNA abundance and tissue-specific distribution (75, 76). Ludwig et al. investigated only tissues derived from male donors, which limits any evaluation of the contribution of the female reproductive tissues to the total miRNA pool found in serum (76). However, all of the miRNAs differentially expressed in our study could be identified in all of the tissues examined by Ludwig et al. with the exception of miR-720, miR-661, and miR-1225-3p (76). A possible explanation as to why miR-720 was not reported could be that it is not a *bonafide* miRNA, but a tRNA fragment (77). When interpreting miRNA profiling results obtained from serum, it is likely that at least some of these may reflect a direct effect from blood cells. It would be very relevant to investigate the tissue source of these miRNAs, which possibly could be done by the capture of exosomes using tissue-specific surface markers.

Packaging of miRNAs into microvesicles, as a way to protect the miRNAs in the circulation but also contributing to intercellular signaling, has emerged as a new and interesting field of study. In this context, circulating platelet-derived microparticles were found elevated in both lean and obese women with PCOS compared with controls (78, 79) and these correlated with serum T. The increased concentration of microparticles has also been associated with HOMA-IR in PCOS subject (80). Moreover, the PCOS associated miR-485-3p was shown to be preferentially packaged into circulating exosomes (81).

Prevalence rates of MetS are higher in women with PCOS compared with the general female population. It is well known that MetS is associated with an increased risk of developing type 2 diabetes and most of these metabolic risk factors are present in PCOS as well. Abnormal levels of miRNAs in relation to MetS or to pathophysiological components hereof have been reported (82–84). Given the public health implications of MetS, early identification is warranted in order to target early interventions. Our three candidate miRNA signature (miR-361-5p, -1225-3p, and miR-34b-3p) had a 97.1% sensitivity and 85.7% specificity for identifying MetS in PCOS. Additionally, serum free T levels are higher in PCOS with MetS compared to PCOS women without MetS (85). Interestingly, serum free T did not increase the AUC, when combined with miRNAs thus there is no significant additional influence of serum free T compared to the 3-miRNA signature alone. We acknowledge that the ROC curve evaluation of the prediction model in the PCOS women was limited by sample size with a lower than expected frequency of MetS in these women, although it still reached statistical significance. Clearly, the discriminatory utility of the 3-miRNA signature should be validated in larger cohorts.

While the clinical usefulness of measuring a circulating miRNA for a PCOS related diagnostic purpose is currently limited, the measurement of miRNAs in a clinical or ambulatory setting using fast microfluidic nanoparticle-based devices is an area of intense research (86) possibly enabling future use of miRNAs for rapid tests. Our study adds novel information to the growing body of evidence indicating a markedly different profile of circulating miRNAs in diseases with elements of metabolic dysfunction (PCOS, impaired glucose tolerance, obesity, non-alcoholic fatty liver disease) (87, 88). Mapping out the miRNAs altered during specific conditions could provide vital cues to identifying their role as biomarkers and/or modulators of the disease process. Future studies will deliver key indications of common and separate pathways to metabolic disease.

In conclusion, we here report comprehensive profiling of miRNAs in serum of PCOS patients and show that PCOS patients have a profile of miRNAs deviating from Controls. Moreover, our data demonstrate that the miRNA profile is specific to subgroups of patients with PCOS: Normoandrogenic PCOS women have circulating miRNAs that cluster differently compared with hyperandrogenic PCOS women. In addition, a three-candidate-miRNA biomarker signature can predict which women among PCOS sub-phenotypes (based on normal and increased free T levels) have a predisposition for developing MetS. It will be relevant to extend these findings to search for miRNAs related to fertility treatment outcomes.

## Data Availability Statement

The raw data supporting the conclusions of this manuscript will be made available by the authors, without undue reservation, to any qualified researcher.

## Ethics Statement

The studies involving human participants were reviewed and approved by Local Scientific Ethical Committee of Region Zealand, DK (approval no. SJ-156). The patients/participants provided their written informed consent to participate in this study.

## Author Contributions

AS, PU, MW, AE, and LD: study conception, design and interpretation of data, and drafting of themanuscript. NS, JG, GM, GJ, RM, AJ, and AH: contribution to figure creation, analysis of data and discussion. All authors read and approved the final manuscript.

### Conflict of Interest

The authors declare that the research was conducted in the absence of any commercial or financial relationships that could be construed as a potential conflict of interest.
