# Supplementary material for: Hyperandrogenism and Metabolic Syndrome Are Associated With Changes in Serum-Derived microRNAs in Women With Polycystic Ovary Syndrome
Source: Front Med (Lausanne). 2019 Nov 1;6:242. doi: 10.3389/fmed.2019.00242 (PMC6839444; doi:10.3389/fmed.2019.00242)
Supplement: Supplementary file 1 [file Data_Sheet_1.docx]

**Supplementary Table 1: Primers for technical validation**

| **miR-484 RT primer** | 5’CTC AAC TGG TGT CGT GGA GTC GGC AAT TCA GTT GAG ATC GGG AG |
| --- | --- |
| **miR-485-3p RT primer** | 5’CTC AAC TGG TGT CGT GGA GTC GGC AAT TCA GTT GAG AGA GAG GA |
| **miR-484 FW primer** | 5’ACA CTC CAG CTG GGT CAG GCT CAG TCC CC |
| **miR-485-3p FW primer** | 5’ACA CTC CAG CTG GGG TCA TAC ACG GCT CT |
| **Universal Reverse Primer** | 5’ TGG TGT CGT GGA GTC G |

**Supplementary Table 2: Highly expressed serum miRNAs within the three patient groups: Control subjects, normoandrogenic PCOS and hyperandrogenic PCOS patients.**

| **miRNA** | **Mean Control**  **(n=20)** | **Mean PCOS**  **(n=42)** | **Mean Normo**  **(n=23)** | **Mean Hyper**  **(n=19)** |
| --- | --- | --- | --- | --- |
| **miR-518f-3p** | 9.5 | 9.8 | 10.0 | 9.5 |
| **miR-518b** | 9.8 | 9.9 | 10.1 | 9.7 |
| **miR-618** | 12.0 | 12.0 | 12.2 | 11.8 |
| **miR-520c-3p** | 12.9 | 12.9 | 13.5 | 12.1 |
| **miR-625-3p** | 13.5 | 13.2 | 13.4 | 12.8 |
| **miR-302c-3p** | 14.0 | 14.3 | 14.4 | 14.1 |
| **miR-1233-3p** | 14.0 | 14.6 | 14.6 | 14.7 |
| **miR-34a-5p** | 14.7 | 14.9 | 14.9 | 14.8 |
| **miR-636** | 16.0 | 16.0 | 16.0 | 15.9 |
| **miR-429** | 19.1 | 18.2 | 18.5 | 17.8 |

Shown are mean raw Ct values for all groups. Normo: Normoandrogenic PCOS patients, Hyper: Hyperandrogenic PCOS patients.

**Supplementary Table 3: The diagnostic utility of individual microRNAs to identify PCOS women with MetS**

| **miRNA** | **AUC** | **p value** |
| --- | --- | --- |
| **miR-20a-5p** | 0.771 | p=0.053 |
| **miR-34b-3p** | 0.767 | **p=0.046** |
| **miR-139-3p** | 0.663 | p=0.297 |
| **miR-361-5p** | 0.917 | **p=0.011** |
| **miR-433-3p** | 0.642 | p=0.283 |
| **miR-485-3p** | 0.576 | p=0.533 |
| **miR-1225-3p** | 0.818 | **p=0.047** |
| **miR-1290** | 0.636 | p=0.267 |
| **miR-21-3p** | 0.580 | p=0.613 |
| **miR-143-3p** | 0.654 | p=0.497 |
| **miR-151-5p** | 0.673 | p=0.191 |
| **miR-155** | 0.589 | p=0.497 |
| **miR-374a-5p** | 0.738 | p=0.071 |
| **miR-378a-5p** | 0.842 | p=0.051 |
| **miR-572** | 0.700 | p=0.310 |
| **miR-638** | 0.842 | p=0.052 |
| **miR-661** | 0.630 | p=0.548 |
| **miR-1227** | 0.714 | p=0.083 |
| **miR-1276** | 0.778 | p=0.361 |
| **miR-720** | 0.682 | p=0.133 |

The diagnostic utility of individual miRNAs in diagnosing metabolic syndrome in PCOS women. MiRNAs displaying significant differences between PCOS subset and controls in ROC analysis, with their corresponding area under the curves (AUCs). ROC curve analysis was only performed in PCOS women. Data were log2 transformed prior to analysis.

**Supplementary Figure 1: Cumulative distribution of different miRNA SD values based on different normalizations strategies.**

Comparison of the cumulative distribution of different normalization strategies to the raw Ct values. SD: Standard deviation. The standard deviations for each individual miRNA were calculated on relative expression levels normalized with global mean normalization (dotted black line) or miR-484 normalization (dotted grey line) approach. The black line represents raw values without normalization.

**Supplementary Figure 2: Correlations among the highest expressed serum miRNAs**

**
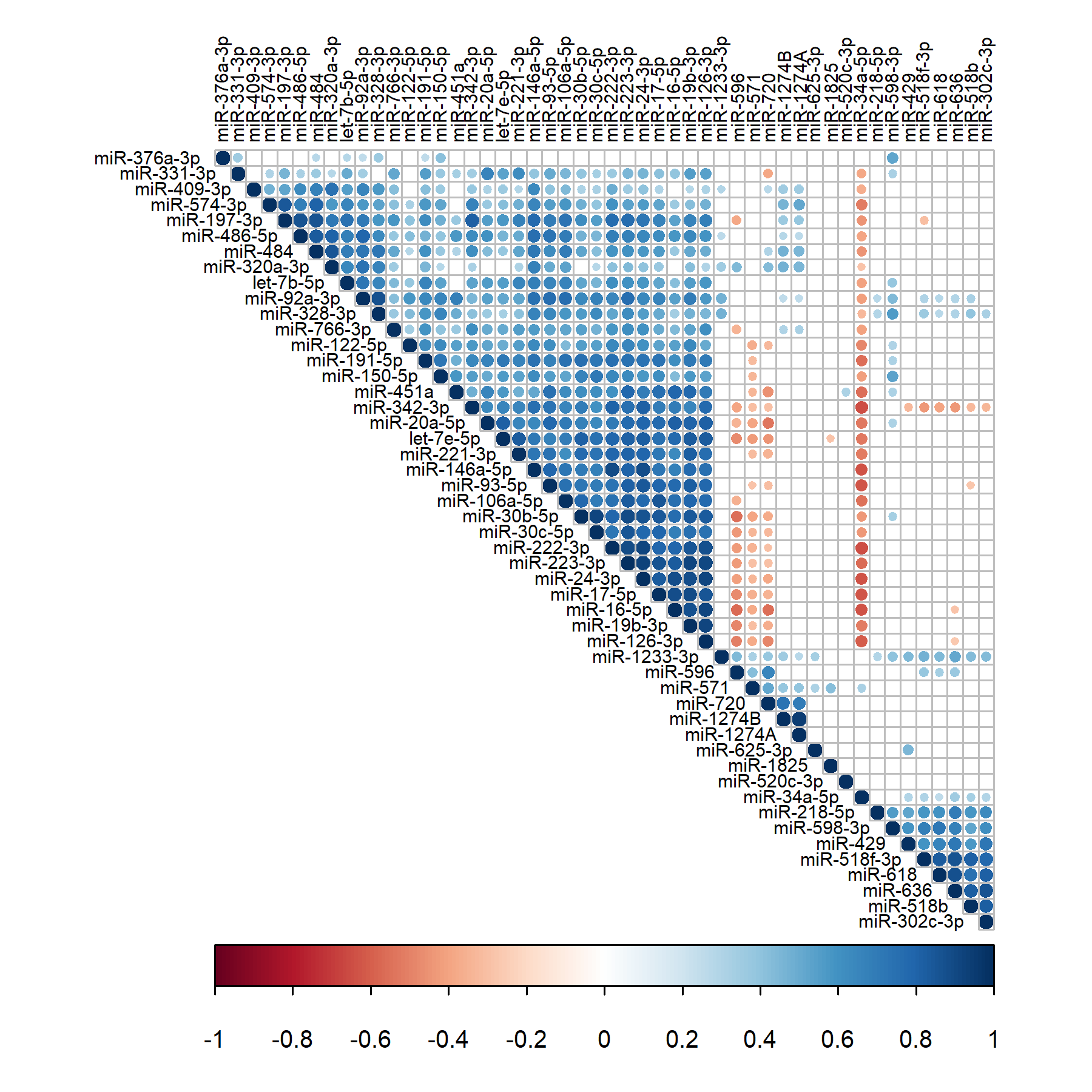
**

Correlation pattern between top 50 abundant serum miRNAs displaying miRNA levels (normalized to the global mean). Color intensity and the size of the circle are proportional to the Pearson correlation coefficient. Positive correlations are displayed in blue and negative correlations in red colors. Indicated correlations are significant at p<0.05.

**Supplementary Figure 3: Inter-subject variability for the entire cohort and the controls.**

Inter-subject variability depicted as rank-plot of coefficients of variation (CV%) for all of the robustly expressed miRNAs (n=303) in all of the samples (A) or controls (B). The twenty differentially expressed miRNAs are marked with open circles while every other miRNA with closed grey circles.

**Supplementary Figure 4: Levels of circulating miRNAs in PCOS patients compared to controls**

Relative fold changes in miRNA quantities normalized to the global mean. Means and SD are displayed. All indicated p-values were determined by Student’s t-test on log_2_ transformed data. *p-value < 0.05. **<0.01.

**Supplementary Figure 5: Technical validation of the array for miR-485-3p by individual qPCR.**

Levels of miR-485-3p relative to miR-484 in PCOS women (n=36) and control subjects (n=17) determined by individual qPCR as technical replication of array data. Indicated p-value was determined by Student’s t-test on log_2_ transformed data. ***p-value < 0.001.

**Supplementary Figure 6: Levels of circulating miRNAs in normoandrogenic and hyperandrogenic PCOS patients compared to controls**

Shown are the relative fold changes of the significantly identified miRNA levels normalized to the global mean and displayed as relative to the control group. All indicated p-values were determined by one-way ANOVA with a post hoc Tukey test on log_2_ transformed data. Data were adjusted for age and BMI. p-value *< 0.05 and **< 0.01. Normo: Biochemical normoandrogenic PCOS, Hyper: Biochemical hyperandrogenic PCOS.

**Supplementary Figure 7: Cluster analysis of circulating miRNAs in different PCOS subsets.**

Heatmap of the 20 miRNAs identified to have significantly different levels in normoandrogenic (Normo) vs. hyperandrogenic (Hyper) PCOS women. Displayed are mean serum miRNA expression level (relative to controls) of each miRNA in either normo- or hyperandrogenic PCOS women.
